# Adamalysines as Biomarkers and a Potential Target of Therapy in Colorectal Cancer Patients: Preliminary Results

**DOI:** 10.1155/2019/5035234

**Published:** 2019-09-02

**Authors:** Katarzyna Walkiewicz, Joanna Strzelczyk, Dariusz Waniczek, Krzysztof Biernacki, Małgorzata Muc-Wierzgoń, Angelika Copija, Ewa Nowakowska-Zajdel

**Affiliations:** ^1^Doctoral Study, School of Medicine with the Division of Dentistry in Zabrze, Medical University of Silesia, Katowice, Poland; ^2^Department of Clinical Oncology, Regional Specialised Hospital No 4, Bytom, Poland; ^3^Department of Medical and Molecular Biology, School of Medicine with the Division of Dentistry in Zabrze, Medical University of Silesia, Katowice, Poland; ^4^Department of Surgery Propedeutics, Chair of General, Colorectal and Trauma Surgery, Medical University of Silesia, Katowice, Poland; ^5^Department and Clinic of Internal Medicine, School of Public Health in Bytom, Medical University of Silesia, Katowice, Poland; ^6^Department of Nutrition Related Disease Prevention, Department of Metabolic Disease Prevention, School of Public Health in Bytom, Medical University of Silesia, Katowice, Poland

## Abstract

Colorectal cancer is one of the most common cancers in the world. Due to its still undetermined pathogenesis, we are searching for signaling pathways that are important in the development of colorectal cancer. In this article, we present results of study on the role of ADAM proteins in colorectal cancer. The study included 85 adult colorectal cancer patients (48 men, 37 women) and 25 patients in the control group (after diagnostic colonoscopy—without cancer). During hospitalization, a serum sample (3 cm^3^) was collected from the study and control group, anthropometric measurements were conducted and others clinical data were analyzed. In the serum ADAM10, 12, 17, and 28, protein concentrations were determined and, in the next step, examined the relationship between ADAMs concentrations and selected clinical parameters in both groups. The analysis showed that serum levels of ADAM10 and ADAM28 are significantly higher in patients with colorectal cancer and correlate with histopathological grading and with presence of distant metastases. Moreover, noticed the trend to correlate concentrations of adamalysines with higher BMI score. One of the functions of adamalysines is the activation of growth factors involved in cancer, including IGF and TNF*α*. The increased activity of adamalysines in patients may play a role in the pathogenesis of colorectal cancer. Our study highlights the prevalence of metabolic disorders in the group of patients with diagnosed CRC, and this cancer seems to be a further complication of obesity.

## 1. Introduction

Colorectal cancer (CRC) is the third most common cancer worldwide. Morbidity mainly affects individuals over 50 years of age. Other epidemiological risk factors include diet (i.e., Western diet with a high, animal-derived fat content and poor in fiber), cigarette smoking, alcohol abuse, and a sedentary lifestyle. These factors altogether often lead to the development of the metabolic syndrome. Obesity, as one of the components of this syndrome, is recognized by WHO as the epidemic of the 21st century and the fifth highest risk factor for death worldwide [1]. Cancer mechanisms associated with the effects of excess body weight are complex. The postulated dependencies include hyperinsulinemia with the activation of insulin-like growth factor (IGF) pathways, co-occurring with obesity, a chronic inflammatory process with the activation of proinflammatory cytokines (TNF*α*, Il-6, and Il-8), monocyte chemoattractant protein (MCP-1), and the promitogenic role of adipose tissue hormones (mostly leptin) [2, 3].

Disorders of insulin balance in the human body affect the functioning of the insulin-IGF-growth hormone (GH) axis. The disturbance of the homeostasis of this axis influences the mitogenic potential of cells, which is also confirmed by epidemiological observations. The analysis of the published cohort and epidemiological studies was conducted by Bardou et al. who confirmed the association between obesity and the development of CRC [4]. Also, in an animal model study, it was shown that TNF*α* expression in colorectal tissue was significantly higher in mice with obesity induced by improper diet than in a group without metabolic disorders [5]. An increase in tumor growth rate due to metabolic disorders induced by a high-fat Western diet was also confirmed by O'Neil et al. High concentrations of leptin, TNF*α*, the presence of visceral white adipose tissue, and hyperinsulinemia were factors that significantly affected the progression of CRC in a mouse model of human CRC [6].

Adamalysines are glycoproteins of diverse structure and common proteolytic function. ADAM—a disintegrin and metalloproteinase, and the term adamalysine are the same term and used to describe the family of the peptidases. In humans, they regulate, e.g., mechanisms of cell migration, adhesion, and bioavailability of growth factors. Each function can play an important role in the development of cancer [7, 8]. For example, it was established that ADAM12 and ADAM28 regulate the pool of free IGF-1 by proteolysis of the IGFBP-3/IGF-1 protein complex [9]. In addition, ADAM17 protein is responsible for the activation of TNF*α*, initiating the signaling pathway associated with the EGF receptor for which it is a ligand, leading to tumor cell proliferation [10]. Herat et al. tried to explain the relationship between ADAM28 and the occurrence of metabolic syndrome. It was established that the expression of ADAM28 was significantly higher in mice with the metabolic syndrome compared to a healthy group. In addition, blocking ADAM28 activity using siRNA technology resulted in body mass index (BMI) reduction, increased insulin sensitivity, and a decreased level of TNF*α* [11]. Another study found that overexpression of ADAM28 in CRC patients occurred both in the tumor tissue and the surrounding surgical margins (histopathologically defined as “clear”). However, the observed overexpression occurred only in overweight or obese patients but not in the normal weight group [12].

These reports show that the role of ADAM family proteins in the pathogenesis of metabolic-dependent cancers, such as CRC, may be significant. In our study, we analyzed serum levels of ADAM10, ADAM12, ADAM17, and ADAM28 in CRC patients, depending on clinical parameters to determine the potential role of adamalysines as a cancer biomarker.

## 2. Materials and Methods

The study group included 85 adult CRC patients (48 men, 37 women) hospitalized in the Department of Internal Medicine, Bytom, Poland. The control group comprised 25 patients who underwent diagnostic colonoscopy during hospitalization without the presence of CRC. During hospitalization, serum samples (3 cm^3^) were collected from all patients. Anthropometric measurements were conducted and the BMI was determined. In addition, other clinical data were analyzed, i.e., the results of biochemical tests, the coexistence of metabolic syndrome components, age, and sex. Patients who had undergone cancer treatment were excluded from the study, as were those with significant immune deficiency, including HCV, HBV, and HIV infections. The study was approved by the Bioethics Committee (no. KNW/0022/KB1/42/14).

The study group was divided based on gender. The characteristics of the study and control groups are shown in Tables 1 and 2.

Serum samples were frozen at 80°C. Assays were performed using ELISA KIT test (Cloud Clone Corporation) in accordance with the producer's recommendations. We used sets of plates with the wells coated with antibodies specific for ADAM10, ADAM12, ADAM17, and ADAM28. We applied biotin-avidin and horseradish peroxidase. Changes in the color of the test solution were measured spectrophotometrically using light with a wavelength of 450 nm ± 10 nm. Concentrations of adamalysines were determined by comparing the results obtained with the results of the standard sample.

The data were processed using Statistica 12. In the first stage of statistical analysis, the relationship between all parameters was examined. Student's *t*-test was used for parameters with normal distribution, and Kruskal-Wallis test was applied for other parameters. ANOVA with post hoc Tukey modification was used to assess the relationship between many independent variables.

Next, the correlation between the examined proteins and the selected clinical parameters was analyzed in the both groups. Correlation analysis was conducted for the study and control groups separately and between these groups. Pearson's correlation coefficient was used for nonlinear correlations, whereas Kendall and Spearman correlation coefficients were used for rank correlation to detect both linear and nonlinear relationships.

## 3. Results

The study and the control groups did not differ in terms of age or BMI (*p* = 0.369 and *p* = 0.946, respectively).

The study group was divided based on the TNM classification, 8th edition. Due to the insufficient number of patients in the study, division into subgroups was not performed. Therefore, the patients from Clinical Stage (CS) I group met the criteria for CSI; those from CSII, the criteria for CSIIA, CSIIB, and CSIIC; patients from CSIII group for IIIA, IIIB, and IIIC; and those from CSIV group for IVA and IVB. The division of the group due to the degree of CRC clinical advancement is presented in Table 3.

Serum concentrations of ADAM10, ADAM12, ADAM17, and ADAM28 obtained from the study and control groups are presented in Table 4.

**Figure 1:**
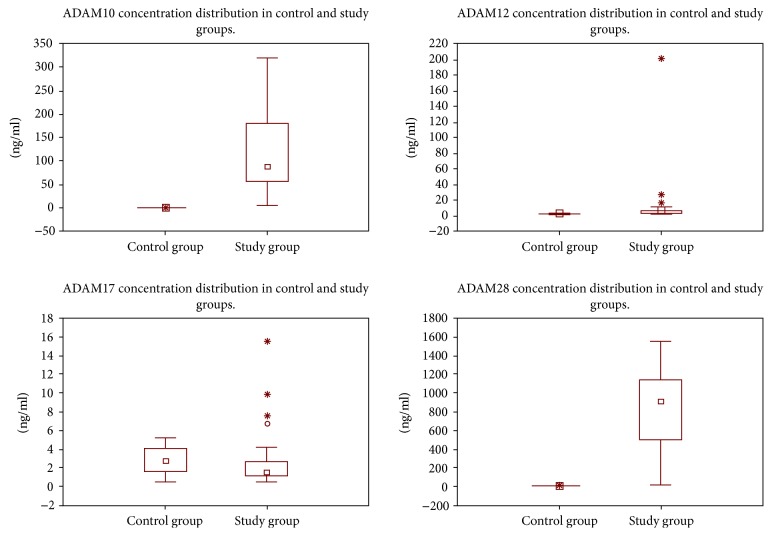
ADAM concentration distribution in both groups.

The maximum concentration values of the proteins (ADAM10 and ADAM28, in particular) found in the study group were observed in many samples. Therefore, they were included in further analyses (Figure 1).

The initial assessment of the concentration values of the ADAMs indicated that the concentrations in the study group were higher than those in the control group. An in-depth statistical analysis revealed that the concentrations in the study group are also dependent on the clinical stage of the disease. The statistical tests showed a relationship between the concentrations of ADAM proteins and the clinical stage of CRC, respectively, for ADAM10, *p* < 0.02, ADAM12, *p* < 0.03, ADAM17, *p* < 0.03, and ADAM28, *p* < 0.005.

**Figure 2:**
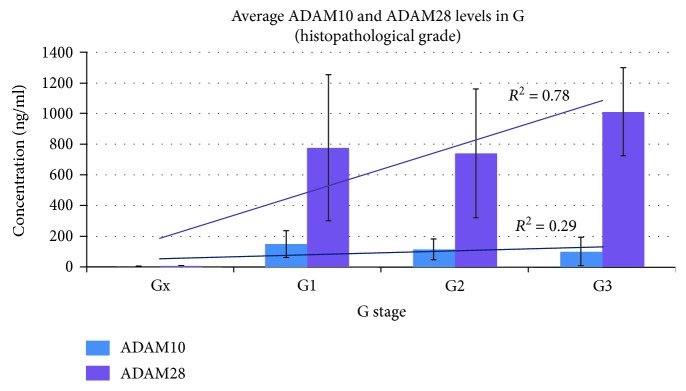
Relationship between serum concentrations of ADAM10 and ADAM28 and histopathological grading.

In addition, a relationship was found between the concentrations of ADAM10 and ADAM28 and histological grading (G1➔G3), the trend line was determined based on the coefficient of determination (*R*^2^), *p* < 0.05 (Figure 2). Moreover, a positive correlation was also found between ADAM28 concentration and the presence of distant metastases (*M*), according to the TNM classification (*p* < 0.05). A similar correlation was not found for other adamalysines.

A positive correlation was found between the CS of CRC and the concentrations of ADAM10 (*p* < 0.001, RHO > 0.35) and ADAM28 (*p* < 0.0001, RHO > 0.53) in the correlation tests. A small negative correlation was found between ADAM17 concentration and CS of the disease (*p* < 0.05, RHO<−0.3).

No significant relationship was observed between concentrations of ADAM10, ADAM12, ADAM17, ADAM28, and BMI. Additionally, no significant corrections were found between these results according to the adopted criteria (*p* < 0.05, RHO > or<±0.3). However, in the group of patients with CRC, values of *p* < 0.05 with RHO = 0.266 were observed for ADAM12 and ADAM17. A similar trend was not found in the control group.

## 4. Discussion

This study is another step in searching for new biomarkers of CRC. The pathogenesis of CRC is complex. Epidemiological data and the results of molecular tests prove the importance of metabolic disorders in the development of CRC [2]. CRC seems to be a complication of obesity with diabetes, impaired renal function, and hypertension. The mechanisms of insulin resistance, hyperinsulinemia, IGF activation, and chronic inflammatory processes participate in the transition from metabolic disorders to malignancy. Selected proteins from the ADAM family play an important role in the activation of this transition. Current data indicate some role of adamalysines in the colon, breast, stomach, prostate, and lung cancer [7, 8, 13].

The present study showed only a trend in the correlation between excess body mass and high concentrations of adamalysines in CRC patients. However, earlier reports showed a possible mechanism of this relationship. ADAM12 and ADAM28 affect the breakdown of the IGF/IGFBP-3 protein complex and simultaneously free release of biologically active growth factors, i.e., IGF1 and IGF2 [9]. The cellular response to IGF is regulated by its binding to IGFBP, and the combination with IGFBP is stronger than the IGF affinity for the receptors. The biologically active IGF1 is one of the strongest growth factors in the activation of the ERK pathway, which is important for the development of CRC [14, 15]. IGFs, especially in association with obesity and insulin resistance, also have an impact on the increased risk of breast cancer [16]. In addition, patients with non-small cell lung cancer (NSCLC) had higher IGF1 and lower IGFBP-3 and IGFBP-7 serum and tumor tissue concentrations compared to patients with noncancerous lung diseases [17]. Similarly, higher cell expression of IGF1 was found in patients with gastrointestinal stromal tumors (GIST) [18]. In contrast, the increase in IGF1R expression in cells with a lower level of IGFBP-3 expression was associated with more advanced pancreatic cancer and worsened the prognosis [19].

*In vitro* tests which confirmed the efficacy of inhibiting further growth of the colon and breast cancer cells by using recombinant IGFBP-3 were also interesting [16, 20]. Furthermore, Nowakowska-Zajdel et al. showed that changes in the expression of ADAM28 and IGFBP-3 were related to the healthy colon tissue in overweight CRC patients. That study showed that the microscopic margin in a group of obese patients was not equal to the molecular margin because changes in gene expression were also observed in normal colon tissue in overweight CRC patients [12]. This observation is very significant for the extent of the surgical treatment and the effectiveness of current treatment, especially in obese patients.

The potential significance of ADAM12 in cancer was reported by Shao et al. who found that ADAM12 was an overexpression in tissue samples of small cell lung cancer, and its expression was correlated with the clinical status. Moreover, serum and urine levels of ADAM12 were higher in patients in an advanced stage of the disease compared to those in the control group [21]. In addition, silencing of ADAM12 expression in H1688 cell lines significantly reduced cellular proliferation, invasion, and metastasis [21]. Other studies showed that ADAM12 could be also a prognostic marker of breast cancer. Roy et al. suggested that ADAM12 overexpression resulted in increased tumor size and metastasis. However, of the two isoforms of ADAM12, only the secreted one enhanced the ability of tumor cells to migrate and invade, resulting in a higher incidence of local and distant metastasis *in vivo*. This stimulatory effect of ADAM12 on migration and invasion was probably dependent on its proteolytic activity. These findings showed that ADAM12 may represent a potential therapeutic target in breast cancer [22, 23]. These observations were confirmed for triple negative breast cancer. Activation of the EGFR/ErbB1/HER1 pathway plays a crucial role in the development of this neoplasm. Li et al. showed that soluble EGF-like ligands were derived from their transmembrane precursors by ADAM proteases and ADAM12 was a protein involved in the proteolysis [24]. The availability of ligands for EGFR depends on the activity of ADAM17 since it was responsible for free TNF*α* formation. Our study showed that serum concentrations of ADAM12 and ADAM17 are closely correlated, which may explain the increased activation of the EGFR pathway at high ADAM12 concentrations.

Analyses of ADAM10 in oral malignancies are also reported. Overexpression of this protein is important in the interaction with the integrin receptor avb6, acting as its ligand, thus increasing tumor invasion and progression [25]. Also in hepatocellular carcinoma (HCC), high levels of ADAM10 protein expression were found, which correlated with disease severity [26, 27]. Moreover, it was shown that ADAM10 may be important in promoting chemoresistance. Resistance to 5-FU and platinum derivatives was observed in patients with advanced metastatic CRC, which could result from altered cell shape due to cell fusion into larger conglomerates. Cell fusion in CRC is promoted, e.g., by ADAM10 [28, 29].

The role of ADAM17 in cancer has been widely reported, especially following the discovery of a direct effect of ADAM17 on the release of TNF*α* [10]. The most recent studies on the role of ADAM17 in CRC found that the cellular level of ADAM17 may augment the malignant potential of CRC cells by increasing their motility and the expression of proangiogenic factors, which could determine tumor progression and metastasis [8]. In another study, it was shown that the interaction between FHL2 and ADAM17 was more frequent in malignant colon tissue compared to normal colon tissue (*p* = 0.005). The mean number of ADAM17/FHL2 proximity ligation assay signals was significantly higher in CRC than in tissues with only low-grade dysplasia. Although FHL2 expression is associated with poor prognosis in CRC, its interaction with ADAM17 in normal, dysplastic, and malignant colon cells may also indicate an adverse role of ADAM17. ADAM17/FHL2 colocalization is more frequent in malignant than in normal or dysplastic cells, suggesting a possible role this protein complex plays in the development/progression of CRC [30]. In turn, other studies focused on the role of environmental factors in the pathogenesis of CRC. It was found that improper Western diet may be a factor that activates the expression of ADAM17. ADAM17 is probably an enzyme induced by Western diet and activated by CXCL12-CXCR4 signaling. The following pathway is postulated: diet→CXCL12→CXCR4→ADAM17→TGF*α*→EGFR activation [31]. High serum concentrations of ADAM17 found in our study in obese patients may be the exponent of the relationship between Western diet and the activation of neoplastic processes. Similar observations are related to higher levels of ADAM17 and TNF*α* among obese patients, which correlate with the severity of atherosclerotic lesions in the blood vessels [32]. An important role of ADAM17 in the pathogenesis of CRC is also confirmed by studies using specific inhibitors of this protein. For example, Rios-Doria et al. showed that the use of a specific anti-ADAM17 antibody (MEDI3622) resulted in the inhibition of the growth of esophageal and CRC cell lines [33]. Similar results were observed by Dosch et al. The use of MEDI3622 resulted in the inhibition of tumor growth in multiple human CRC PDX models and also improved the survival of animals bearing tumor xenografts. Those authors demonstrated that MEDI3622 was further found to impact the Notch pathway activity and tumor-initiating cells [34].

A significantly increased risk of developing CRC with metabolic disorders, such as those discussed above, indicates the need to implement preventive measures. Modifiable risk factors of a high-fat Western diet and a sedentary lifestyle include obesity and carbohydrate metabolism disorders. The prophylactic effect related to the decrease in the risk of developing CRC has already been confirmed for, e.g., flavonoids in green tea, curcumin, and carotenoids. This role is also postulated for some drugs, including acetylsalicylic acid, metformin, and some statins [35].

In light of this research, possibilities for designing therapies targeted at key points during colon cancer development are of crucial importance. These therapies could be related to adamalysines and the activation of signaling pathways for IGF and EGF. The potential value of this study is already evidenced by the results of the beneficial effects of using specific inhibitors for adamalysines. The use of the ADAM concentration in the early diagnosis and the follow-up of cancers is of particular importance. However, further studies are warranted on larger samples to objectify the results.

Our study needs to be viewed in light of its limitation. The serum concentration of ADAMs seems to be results not only of cancer. The study group was not homogeneous enough. The authors wanted to continue research on a larger group and study of protein expression in tissue to confirm obtained results.

## Figures and Tables

**Table 1:** Characteristics of the study group.

Gender	Age			BMI (kg/m^2^)			Overweight (BMI value: 25.0-29.9), *n* [%]	Obesity (BMI value: >30.0), *n* [%]
	Min	Max	Mean	Min	Max	Mean		
Women (*n* = 37)	47	86	69.7	19.5	34.3	26.4	12 [32.4]	9 [24.3]
Men (*n* = 48)	32	88	68.1	18.2	35.9	26.4	21 [44.7]	4 [17.0]
All (*n* = 85)	**32**	**88**	**68.8**	**18.2**	**35.9**	**26.4**	**33 [38.6]**	**13 [15.3]**

**Table 2:** Characteristics of the control group.

Gender	Age			BMI (kg/m^2^)			Overweight (BMI value: 25.0-29.9), *n* [%]	Obesity (BMI value: >30.0), *n* [%]
	Min	Max	Mean	Min	Max	Mean		
Women (*n* = 14)	36	86	70.9	20.1	30.1	26.6	7 [53.3]	1 [7.1]
Men (*n* = 11)	54	85	71	21.2	33.4	26.4	4 [36.4]	2 [18.2]
All (*n* = 25)	**36**	**86**	**71**	**20.1**	**33.4**	**26.5**	**11 [44.8]**	**3 [12.6]**

**Table 3:** Division of the study group depending on the clinical stage of the disease.

Gender	CSI	CSII	CSIII	CSIV	Histopathological grade			Localization	
					G1	G2	G3	Colon	Rectum
Female (*n* = 37)	2	14	13	8	6	19	5	32	5
Men (*n* = 48)	3	15	14	16	6	28	7	32	16
All (*n* = 85)	**5**	**29**	**27**	**24**	**12**	**47**	**12**	**64**	**21**

**Table 4:** Serum concentrations of ADAM10, ADAM12, ADAM17, and ADAM28 in both groups.

Clinical staging	ADAM10 (ng/ml)			ADAM12 (ng/ml)			ADAM17 (ng/ml)			ADAM28 (ng/ml)		
	Min	Max	Mean	Min	Max	Mean	Min	Max	Mean	Min	Max	Mean
CSI	1.7	260.7	**140.9**	0.6	26.7	**5.4**	0.65	9.8	**2.4**	244.8	1226.3	**774.8**
CSII	9.9	270.7	**104.3**	0.8	20.7	**3.4**	0.4	15.5	**1.5**	21.8	1545.8	**835.0**
CSIII	20.5	256.3	**101.4**	1.9	16.4	**4.52**	0.9	7.6	**1.8**	17.1	1251.8	**619.4**
CSIV	79.4	321.8	**111.9**	2.6	15.8	**5.69**	0.8	3.8	**2.33**	479.6	1422.3	**1019.7**
Control group	0.03	1.14	**0.32**	0.26	3.81	**1.99**	0.71	5.25	**2.78**	1.67	14.36	**3.72**

## Data Availability

The data used to support the findings of this study are available from the corresponding author upon request.

## Abbreviations

ADAM: A disintegrin and metalloproteinase
CAF: Cancer-associated fibroblasts
CEA: Carcinoembryonic antigen
CS: Clinical stage
CTGF: Connective tissue growth factor
CXCL1-2: Chemokine (C-X-C motif) ligand1-2
EGF: Epidermal growth factor
ELISA: Enzyme-linked immunosorbent assay
FGF: Fibroblast growth factor
FHL-2: Four and a half LIM domains protein 2
HER: Human epidermal growth factor receptor
HRP: Horseradish peroxidase
IGF: Insulin-like growth factor
IGFB: Insulin-like growth factor binding protein
IR: Insulin receptor
MMP: Matrix metalloproteinase
NSCLC: Non-small cell lung cancer
Rho: Spearman's rho correlation
R^2^: Coefficient of determination *R*^2^
TGF*β*: Transforming growth factor *β*
TIMP: Tissue inhibitors of metalloproteinase
TNF*α*: Tumor necrosis factor *α*
VEGF: Vascular endothelial growth factor.
